# The José Planellas Herbarium (Herbarium BCN)

**DOI:** 10.3897/BDJ.14.e174025

**Published:** 2026-02-18

**Authors:** Roser Guardia, Ignasi Soriano

**Affiliations:** 1 Plant Biodiversity Reource Centre of the University of Barcelona la UB, Barcelona, Spain Plant Biodiversity Reource Centre of the University of Barcelona la UB Barcelona Spain; 2 University of Barcelona, Barcelona, Spain University of Barcelona Barcelona Spain

**Keywords:** occurrence, historical collections, Flora of Galicia, vascular plants

## Abstract

**Background:**

This data paper presents the José Planellas Herbarium, a historic collection that served as the basis for the publication of the first systematic flora of Galicia, Spain.

**New information:**

The Planellas Herbarium includes 2,483 specimens, of which 1,654 have locality data and have been registered in GBIF. The Planellas’s herbarium contains specimens of 1,922 taxa at specific or infraspecific level (subspecies) belonging to 835 genera in 142 families. The most abundant families are Asteraceae, Poaceae and Lamiaceae. The specimens were manly from Galicia, in Spain. Moreover, this herbarium contains plants from other countries, mainly Germany and France, that have handwritten labels written by Willkomm with whom Planellas appears to have maintained specimen exchange. Most of the samples were collected before 1852, when he was a professor at the University of Santiago de Compostela. This Herbarium hosts the specimens that served as the basis for the preparation of the first systematic flora of Galicia (Spain), including nine types.

## Introduction

The Herbarium of the University of Barcelona (Spain) is in the Diagonal campus of the City of Barcelona. It holds more than 400,000 specimens, which are testimony to the research carried out at the University of Barcelona in the field of Plant Biodiversity from the 19^th^ century to the present day. The Herbarium is registered in the *Index Herbariorum* with the acronym BCN. It includes specimens of all the major botanic groups: algae, bryophytes, vascular plants, fungi and lichens. The oldest herbarium specimens, from 19^th^ century, are mainly kept separately. Historical collections require special handling, which is more labour-intensive and a considerable portion (around 10,000 specimens) has not yet been fully processed conveniently.

One of these ancient collections is the Herbarium of José Planellas Giralt (1820-1888), who was a professor at the University of Barcelona (Barcelona, Catalonia) from 1868 until his death in 1888. Planellas was born in Barcelona in 1820. Between 1838 and 1846, he pursued studies in Philosophy, Chemistry applied to the arts and Medicine and Surgery. After completing his academic training, he was appointed professor at the Institute of Jaén (Jaén Andalucia) in 1846. Just one year later, he was awarded the Chair of Natural History in the Faculty of Philosophy at the University of Santiago de Compostela (A Coruña, Galicia). In 1854, he earned a degree in Natural Sciences from the same university. He remained in Santiago until 1868, when he returned to Barcelona to assume a similar position at the University of Barcelona.

From his earliest years in Santiago, Planellas conducted floristic surveys, primarily in the areas surrounding the city and other parts of the northwest of Spain. These expeditions marked the beginning of the herbarium examined in this study. In 1852, he published his main work, *Ensayo de una flora fanerogámica gallega* (Essay on a Galician Phanerogamic Flora; Planellas 1852; hereafter *Flora Gallega*), the first systematic flora of Galicia. In this work, he recorded 853 taxa with their medicinal uses and described 12 new taxa (Guardia and Soriano 2016).

Planellas maintained contact with prominent foreign botanists of his time, particularly with the two authors then working on *Prodromus Florae Hispanicae* (Willkomm and Lange 1861), one of the earliest comprehensive flora of the Iberian Peninsula (Spain and Portugal). This flora includes more than 540 references to Planellas’s specimens. M. Willkomm (1821-1895) even named a species in his honour: *Dianthus planellae* Willk. The Danish botanist Johan Lange (1818-1898) met Planellas during a botanical campaign in Galicia in 1852 and provided him with a substantial amount of his own unpublished data and some herbarium specimens.

As previously noted, Planellas returned to Barcelona in 1868 to join the Faculty of Sciences at the University of Barcelona. He obtained a doctorate in Natural Sciences from the same university in 1869. He served as acting rector in 1880 and held the position of Dean of the Faculty of Sciences until his death in 1888. He was also a full member of the Royal Academy of Sciences and Arts of Barcelona.

When Planellas moved to Barcelona in 1868, he brought with him the main part of his herbarium, which is now hosted in BCN Herbarium. This paper describes the Herbarium of the J. Planellas, published through GBIF portal in 2025 ().

## General description

### Purpose

**Figure 1.**
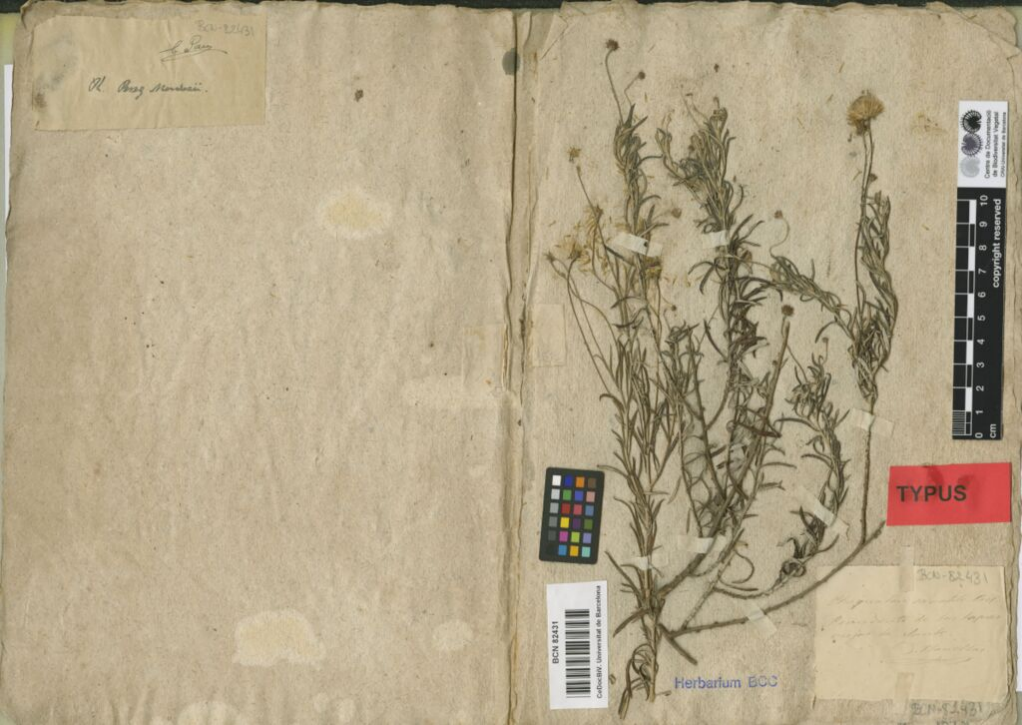
Typical format of a sheet from the Planellas Herbarium.

The Herbarium of Planellas contains mainly flowering plants, but also includes some cryptogamic specimens. However, these latter specimens are neither identified nor have their locality of origin indicated and, therefore, have not been included in the dataset. As for the vascular plants, most of them are mounted on sheets smaller than the standard size (31 x 21 cm), often consisting of two or three separate sheets each (Fig. 1).

A total of 2,483 specimens have been processed and included in the herbarium database. The information provided on the original labels was rather limited, as is common in many 19^th^-century historical collections. They usually include the scientific name and the locality, but neither the collector nor the collection date is indicated. By studying Planellas’s handwriting on the labels where he is listed as the collector, it has been possible to deduce with confidence that more than 80% of the specimens were collected by him. Of the remaining specimens in the Herbarium, 216 have labels written by Pérez Méndez, a disciple of Planellas who appears to have assisted him in organising the Herbarium. These labels are generally more detailed than those of Planellas, typically including the year of collection (199 specimens) and habitat information (194 specimens), although the collector is rarely specified (only five specimens). These Pérez Méndez specimens date from 1866 and 1867 and are, thus, postdated to the publication of the *Flora Gallega*.

Additionally, there are 136 specimens labelled by M. Willkomm, with equally comprehensive labels. At least ten different botanists are cited as collectors, including Willkomm himself. These specimens were dated between 1828 and 1856 and are originally from various Central European localities. Planellas exchanged herbarium specimens with Willkomm, as confirmed by the collections held in both their herbaria. The Willkomm Herbarium conserved in Coimbra contains at least 17 herbarium sheets collected by Planellas.

Six specimens list Gil as the collector, referring to J. M. Gil Rey (1815–1853), a physician who briefly taught natural history at the University of Santiago de Compostela. As cited by Planellas, Gil, in collaboration with T. Martínez Servida, initiated the formation of a herbarium in 1846, which they envisioned as the foundational material for compiling a flora of Galicia (Gil Rey and Martínez Servida 1849).

Finally, four specimens are written in different handwriting and cite J. Lange as the collector. These four specimens, all from Galicia and dated September 1852, correspond to J. Lange’s visit to the region, during which he is known to have interacted with Planellas.

A distinctive feature of Planellas' *Flora* is the inclusion of cultivated plants, as explicitly stated in the introduction to this work. His herbarium contains at least 695 specimens of cultivated plants. In 308 of these, the labels clearly indicate their cultivated status. Most of these specimens were collected in the Santiago Botanical Garden (Santiago de Compostela, A Coruña, Galicia), also known as the Garden of Fonseca (115 specimens) and the Barcelona Botanical Garden (Catalonia, 47 specimens).

## Sampling methods

### Sampling description

All the specimens of the Planellas Herbarium have been revised carefully. The information contained in the herbarium labels is very heterogeneous and rather scarce, but it has been reviewed and supplemented with different sources of information whenever possible.

The herbarium labels are handwritten. Their handwriting has been carefully studied and, in most cases, it has been possible to attribute it to a specific person.

The localities often refer to places that currently have different names or have changed their administrative affiliation. All specimens lack geographic coordinates. In all cases where it has been possible, their current name has been identified. The geographical coordinates have been obtained using the Vissir tool of the Cartographic Institute of Catalonia (http://srv.icgc.cat/vissir3/) and the Iberpix tool of the Spanish National Geographic Institute (https://www.ign.es/iberpix/visor/). When locality names refer to large areas (such as a province, a large city or a massif), we designed the geographical coordinates of the central point of that area and, as uncertainty, we used a value in metres equal to the radius of the smallest circle that encompasses the entire area represented by that name. It was not possible to geolocalise data from labels that include more than one locality or from administrative divisions that no longer have a present-day equivalent.

All data have been entered into a MS-Access relational database designed for herbarium BCN digitisation by its curators. The dataset was published using the Integrated Publishing Toolkit (IPT) hosted by GBIF Spain (https://www.gbif.org/es/ipt).

### Quality control

A standardisation and homogenisation of data have been undertaken using the tools offered by the Global Biodiversity Information System (GBIF). The occurrence data were accommodated to fulfil the Darwin Core Standard (Wieczorek et al. 2012). Scientific names has been checked with the tool Species matching of the GBIF labs. The indication of the country and province has been standardised following the ISO 3166.

The Darwin Core Archive file was uploaded to the IPT (Integrated Publishing Toolkit, IPT) hosted by GBIF.ES. The metadata from the dataset have been completed directly in the IPT.

## Geographic coverage

### Description

The 1.654 specimens with locality where manly from Spain, although other countries are also represented (Table 1).

All the specimens from outside Spain were collected by other botanists and include handwritten labels written by Willkomm with whom Planellas appears to have maintained an exchange of herbarium specimens. These specimens are originally from various Central European localities: France, Germany, Switzerland and Portugal.

**Figure 2.**
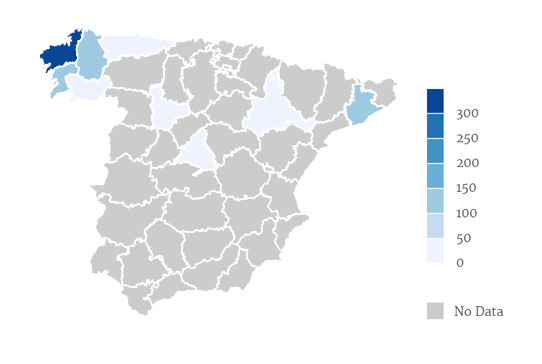
Number of specimens by Spanish province.

Amongst the records from Spain, most of the specimens were collected through the four Galician provinces (A Corña, Lugo, Orense and Pontevedra, mainly from A Coruña). A few plants were collected from Barcelona (Catalonia), Madrid or Oviedo (Asturias) amongst others (Fig. 2).

## Taxonomic coverage

### Description

Almost all specimens in the Planellas Herbarium sheets (99.76%) contained a label with a proper identification, most likely assigned by Planellas himself. Since the Herbarium was transferred to the University of Barcelona — brought there by Planellas himself upon his appointment as professor — the specimens have been revised taxonomically by taxonomic specialists. The most comprehensive revision was carried out around 1920 by the botanist C. Pau (1857–1937), who published his findings in a series of four articles in the journal *Brotéria* (Pau 1921a, Pau 1921b, Pau 1922, Pau 1924). There are 163 herbarium sheets with revision labels from Pau, where he confirms or corrects the identification made by Planellas. Some of the specimens Pau had on loan were ultimately incorporated into his personal herbarium, which was later transferred to the MA Herbarium of the Real Jardín Botánico de Madrid (Soriano and Guardia 2016).

Other botanists who have examined this Herbarium include M. Lainz (1923–2024), B. Merino (1845–1917) and, more recently, C. Aedo (1960–). Finally, I. Soriano, one of the present authors, has re-examined all the specimens in the Herbarium according to current taxonomic criteria.

**Figure 3.**
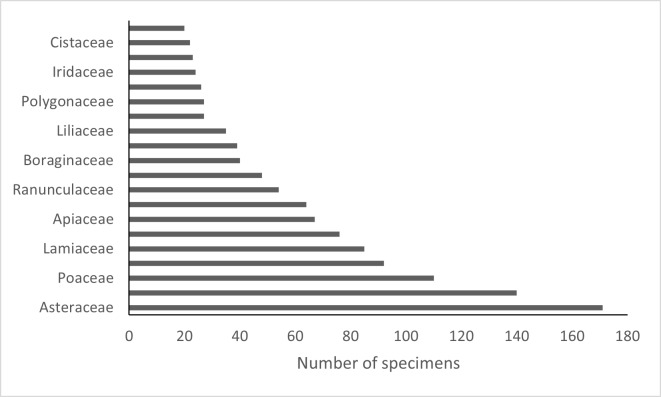
Number of specimens by family. Only families with more than 20 sheets have been represented.

The Planellas’s herbarium contains specimens of 1,922 taxa at specific or infraspecific level (subspecies) belonging to 835 genera in 142 families. The most abundant families are Asteraceae, Poaceae and Lamiaceae (Fig. 3). The number of taxa represented in the Herbarium far exceeds the 853 taxa listed in *Flora Gallega*. In 1858, Planellas participated in the "Exposición Agrícola, Industrial y Artística de Galicia", where he exhibited a herbarium comprising 891 taxa and compiled the catalogue for the entire exhibition (Planellas et al. 1858). In addition to his own herbarium, six other herbaria were displayed at the exhibition — three consisting of cultivated plants and the remainder mainly of spontaneously growing flora. It is possible that Planellas incorporated some of these specimens into his own herbarium without indicating their origin.

## Temporal coverage

**Data range:** 1828-1-01 – 1890-12-31.

### Notes

**Figure 4.**
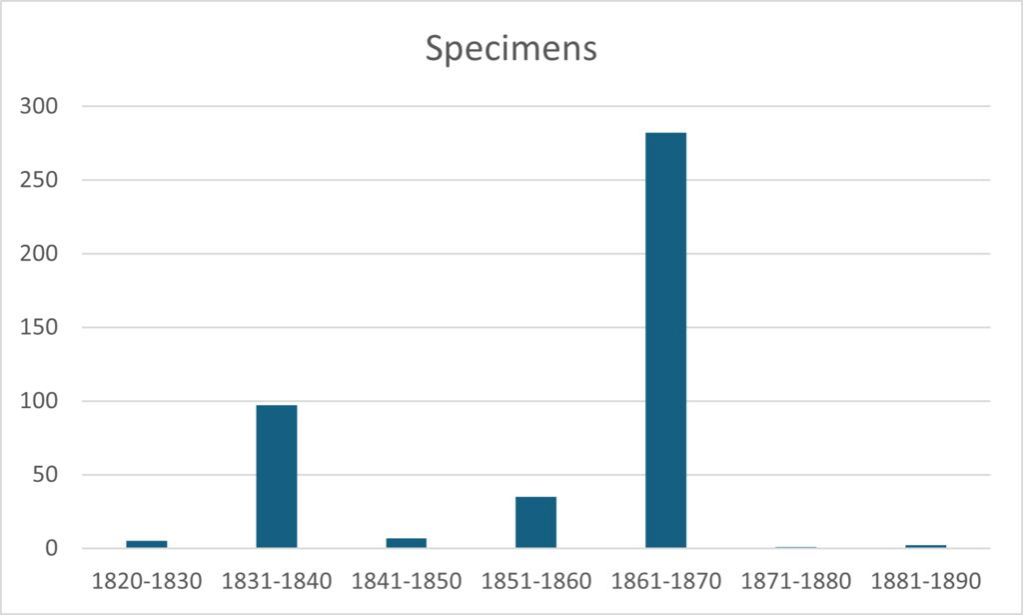
Chronological distribution of Planellas's Herbarium specimens by year.

Only 434 sheets in the Planellas Herbarium contain information regarding the date of collection. It is highly probable that most of the specimens were collected prior to 1852, the year in which *Flora Gallega* was published, as the localities listed in that work are largely consistent with those indicated on the herbarium labels (Fig. 4).

Amongst the dated specimens, only 89 sheets (20.75%) list Planellas as the collector or bear labels that appear to have been prepared by him, although his name is not explicitly mentioned. Two of these specimens are from Madrid, dated 1846; eleven are from Galicia and were collected between 1860 and 1872; the remainder were all collected in Catalonia between 1863 and 1885.

Of the remaining dated specimens, 187 sheets (43.59%) include labels prepared by Pérez Méndez and are dated 1866 and 1867, all originating from Galicia. The final 134 sheets (31.24%) were received from the botanist Willkomm and are dated between 1828 and 1856.

## Usage licence

### Usage licence

Open Data Commons Attribution License

### IP rights notes

This work is licensed under a Creative Commons Attribution Non Commercial (CC-BY-NC 4.0) License.

## Data resources

### Data package title

CeDoc de Biodiversitat Vegetal BCN Herbari J. Planellas

### Resource link

https://doi.org/10.15470/0jebqb

### Alternative identifiers

https://www.gbif.org/dataset/9b5fee02-2ffd-4cbf-88b5-25fe35a0bc37

### Number of data sets

1

### Data set 1.

#### Data set name

CeDoc de Biodiversitat Vegetal BCN Herbari J. Planellas

#### Data format

Darwin Core

#### Download URL

https://www.gbif.org/dataset/9b5fee02-2ffd-4cbf-88b5-25fe35a0bc37

#### Description

The Planellas Herbarium includes 2,483 specimens, of which 1,654 have locality data and have been registered in GBIF (Guardia 2025). The Planellas’ Herbarium contains specimens of 1,922 taxa at specific or infraspecific level (subspecies) belonging to 835 genera in 142 families. The most abundant families are Asteraceae, Poaceae and Lamiaceae.

**Data set 1.** 

Column label	Column description
scientificName	The full scientific name with authorship.
scientificNameAuthorship	The authorship information for the scientific name.
basisOfRecord	The specific nature of the data record, always preserved specimen.
catalogNumber	The number of herbarium of the specimen in BCN Herbarium.
class	The full scientific name of the class in which the dwc:Taxon is classified.
collectionCode	The acronym identifying the collection of Cormophyta of the BCN Herbarium.
coordinateUncertaintyInMeters	The radius, in metres, of the smallest circle centred on the dwc:decimalLatitude and dwc:decimalLongitude point.
country	The name of the country in Spanish.
countryCode	The name of the country with the ISO 3166-1 code.
modified	Date on which the resource was changed.
datasetName	Name of the dataset: Herbari J. Planellas.
dateIdentified	The date on which the subject was determined conforming to ISO 8601-1:2019.
day	The day of the month on which the specimen was collected.
decimalLatitude	The geographic latitude in decimal degrees, using the geodetic datum WGS84.
decimalLongitude	The geographic longitude in decimal degrees, using the geodetic datum WGS84.
family	The full scientific name of the family in which the Scientific name is classified.
genus	The full scientific name of the genus in which the Scientific name is classified.
geodeticDatum	The geodetic datum upon which the geographic coordinates are based: WGS84.
georeferencedBy	A list of names of people who determined the georeference data of the location.
habitat	Description of the habitat in which the specimen was collected as described by the collector.
identificationQualifier	The taxonomic level where the determiner express doubts preceded by the term cf.
identificationRemarks	Comments expressed by the determiner about the Identification.
identifiedBy	A list of names of people who assigned the Scientific name to the specimen.
datasetID	The doi of the set of data.
infraspecificEpithet	The name of the lowest infraspecific epithet of the Scientific name, excluding any rank designation.
institutionCode	The acronym of the Herbarium of the University of Barcelona BCN), where the specimens were kept.
kingdom	The full scientific name of the kingdom in which the Scientific name of the specimen is classified.
language	The code of the language of the resource (es).
year	The four-digit year in which the specimen was collected.
locality	The toponym of the place where the plant was collected.
minimumElevationInMeteres	minimum ElevationInMetres
	
month	The month on which the specimen was collected.
occurrenceID	An identifier that consists of for the dwc:OwnerInstitutionCode: dwc:collectionCode: dwc:catalogNumber.
occurrenceRemarks	Comments about the occurrence that cannot be included in any other tag.
	
order	The full scientific name of the order in which the scientific name is classified.
ownerInstitutionCode	Code of Institution owner.
phylum	The full scientific name of the phylum in which the scientific name is classified.
previousIdentifications	A list of previous assignments of names to the specimen.
recordedBy	A list of names of people that collected the specimen.
verbatimLocality	The original textual description of the place.
specificEpithet	The name of the species epithet of the scientific name.
stateProvince	The name of the province in which the locality occurs.
type	The nature or genre of the resource.
taxonRank	The taxonomic rank of the most specific name of the scientific name.
institutionID	The identifier of the BCN Herbarium from GBIF.
typeStatus	The nomenclatural type that defines the specimen.
license	A link to the Creative Commons permission specifyng what is possible to do with the dataset.
eventDate	The date when the specimen was collected.
verbatimEventDate	The original date as it appears on the label of the specimen.

## Figures and Tables

**Table 1.** Number of specimens by country.

**Country**	**Specimens**
Spain	1,506
France	66
Germany	53
Switzerland	2
Portugal	1
